# Pediatric Laryngopharyngeal Reflux: An Evidence-Based Review

**DOI:** 10.3390/children10030583

**Published:** 2023-03-18

**Authors:** Jerome R. Lechien

**Affiliations:** 1 Polyclinic of Poitiers, Elsan Hospital, 86000 Poitiers, France; jerome.lechien@umons.ac.be; Tel.: +32-65-37-35-84; 2 Department of Anatomy and Experimental Oncology, Mons School of Medicine, UMONS Research Institute for Health Sciences and Technology, University of Mons (UMons), B7000 Mons, Belgium; 3 Department of Otolaryngology-Head and Neck Surgery, EpiCURA Hospital, B7000 Baudour, Belgium; 4 Department of Otolaryngology-Head and Neck Surgery, EpiCURA Hospital, Rue L. Cathy, University of Mons, B7000 Mons, Belgium

**Keywords:** larynx, laryngitis, laryngopharyngeal, reflux, otolaryngology, head neck surgery, gastroesophageal reflux, infants, children, pediatric

## Abstract

Purpose: Pediatric laryngopharyngeal reflux (P-LPR) is associated with the development of common otolaryngological symptoms and findings. In the present study, the findings about epidemiology, clinical presentation, diagnostic and therapeutic outcomes of pediatric population were reviewed. Methods: A PubMed, Cochrane Library, and Scopus literature search was conducted about evidence-based findings in epidemiology, clinical presentation, diagnostic and therapeutic outcomes of P-LPR. Findings: The prevalence of LPR remains unknown in infant and child populations. The clinical presentation depends on age. Infants with LPR symptoms commonly have both gastroesophageal reflux disease (GERD) and laryngopharyngeal reflux and related digestive, respiratory and ear, nose and throat symptoms. The GERD prevalence appears to decrease over the growth, and the clinical picture is increasingly associated with LPR symptoms and findings without GERD. The prevalence of LPR and proximal acid and nonacid esophageal reflux events may be high in some prevalent otolaryngological conditions (chronic otitis media, laryngolomalacia and apnea). However, the lack of use of hypopharyngeal–esophageal multichannel intraluminal impedance pH monitoring (HEMII-pH) limits the establishment of etiological associations. Proton pump inhibitors are less effective in P-LPR patients compared to GERD populations, which may be related to the high prevalence of weakly or nonacid reflux events. Conclusions: Many gray areas persist in P-LPR and should be not resolved without the establishment of diagnostic criteria (guidelines) based on HEMII-pH. The unavailability of HEMII-pH and the poor acid-suppressive therapeutic response are all issues requiring future investigations. Future controlled studies using HEMII-pH and enzyme measurements in ear, nose or throat fluids may clarify the epidemiology of P-LPR according to age and its association with many otolaryngological conditions.

## 1. Introduction

The backflow of gastric content into the esophagus is a physiologic process occurring in most infants and children without complication [1]. When the reflux leads to troublesome symptoms and/or complications, such as esophagitis or stricturing, the gastroesophageal reflux becomes pathologic and is defined as gastroesophageal reflux disease (GERD) [1,2]. Reflux disease may be associated with upper aerodigestive tract symptoms and findings, leading to the use of the terms “pediatric extra-esophageal reflux disease” or pediatric laryngopharyngeal reflux (P-LPR) [3]. Although LPR was considered as an extension of GERD, the differences in clinical presentation and therapeutic outcomes led to the evolution of P-LPR as a distinct disease process [4]. From a clinical standpoint, symptoms and findings of P-LPR are known to vary widely by age and remain non-specific [3,4]. P-LPR may be associated with many laryngeal, oral, nasal or ear conditions, e.g., otitis media, chronic rhinosinusitis or dysphonia, even if proving causality remains difficult in most of them [5,6]. The diagnosis of P-LPR remains complicated regarding the non-specificity of symptoms and signs, and the difficulty to make a pH study in pediatric population [7,8]. Thus, infants and children represent a challenging population. 

The aim of this evidence-based review was to report the current knowledge about epidemiology, clinical picture, diagnostic and therapeutic outcomes of infants and children with laryngopharyngeal reflux disease and to provide critical evidence-based analysis of the current literature. 

## 2. Materials and Methods

A PubMed, Cochrane Library and Scopus database search was conducted for relevant peer-reviewed publications in English and French related to incidence, prevalence, symptoms, signs, pathophysiology, diagnosis and treatment of P-LPR. The following key words were associated: “pediatric”, “children”, “infant”, “reflux”, “laryngopharyngeal”, “extra-esophageal”, “gastroesophageal”, “symptoms”, “findings”, “treatment” and “physiology”. Clinical prospective/retrospective controlled/uncontrolled studies, systematic reviews or meta-analyses were considered in the review process. The papers were selected if they provided data on epidemiology, pathophysiology, clinical presentation, diagnosis and treatment of LPR in pediatric population. Critical analysis of the included publications was carried out focusing on incidence and prevalence, clinical presentation, diagnosis and treatment of infants and children with LPR. The author summarized implications for practice. Ethics committee approval was not required for this state-of-the art review. A systematic review or meta-analysis was not performed regarding the low number of studies and the heterogeneity across studies in inclusion/exclusion criteria, pH-testing use, treatment and therapeutic outcomes. 

## 3. Epidemiology

The prevalence of GERD-related symptoms in the pediatric population varied from 2% to 30% in Western countries [9,10] and appears to increase with the increase in the incidence of the childhood obesity [4]. Recent studies supported that GERD occurs in 50% of infants younger than 2 months of age, 60–70% of infants 3–4 months and 5% of infants of more than 12 months of age [11,12,13].

To date, both the prevalence and incidence of P-LPR are still unknown, because there was no investigation of prevalence or incidence in the pediatric population through objective diagnostic tools. At best, the prevalence of reflux was investigated in infants or children with upper airway symptoms who were addressed in pediatric otolaryngology consultations [14]. The prevalence of acid esophageal reflux events in infants and children with apnea or stridor ranged from 27% to 73% [15,16,17]. Among pediatric patients with chronic cough or hoarseness, GERD was detected in 62% to 73% of cases [17,18,19]. The prevalence of P-LPR in upper respiratory diseases and symptoms remains, however, uncertain, because the authors of these studies determined the presence of reflux through single or dual-probe pH monitoring, which cannot detect weakly or alkaline pharyngeal reflux events. 

From an evidence-based approach, the lack of use of hypopharyngeal–esophageal multichannel intraluminal impedance pH monitoring (HEMII-pH) makes difficult the determination of the exact prevalence and incidence of P-LPR in infants and children. The determination of P-LPR prevalence or incidence requires the use of HEMII-pH or oropharyngeal pH testing, which are the only two approaches able to detect acid, weakly acid and alkaline pharyngeal reflux events [20,21]. Moreover, to date, there have been no international guidelines about the cutoff for the P-LPR diagnostic with HEMII-pH. The future determination of P-LPR incidence and prevalence should consider the age of children/infants because as with GERD, P-LPR is expected to be more prevalent during the first 12 months of life according to the immaturity of esophageal sphincters [22]. 

## 4. Pathophysiology 

### 4.1. Physiology of Laryngopharyngeal Reflux 

Regardless of the patient’s age, the development of laryngopharyngeal symptoms and findings may be attributed to the deposit of gastroduodenal content (e.g., pepsin, bile salts) into the upper aerodigestive tract mucosa and the related development of an inflammatory reaction [23,24]. The occurrence of pharyngeal reflux events may consist of a liquid backflow of stomach content, especially in infants with regurgitations, or it may be gaseous and silent in children without regurgitation [15,25]. The P-LPR appears to be more likely weakly acid at the HEMII-pH, which is substantially different from the GERD profile at the HEMII-pH [7,15]. Despite the lack of study investigating the P-LPR features at the (HE)MII-pH according to the age, the presence of esophageal immaturity in young infants should be associated with the co-existence of GERD and different HEMII-pH tracings and features. In other words, P-LPR characteristics may vary according to the age of the patient. 

The inflammatory reaction in upper aerodigestive tract mucosa may lead to mucosal injuries, mucus dryness, epithelium thickening and micro-trauma [26]. The mucosa injuries lead to mucus production and dehydration through a down-regulation of mucin and carbonic anhydrase gene expression [26]. Basic science and adult clinical studies reported that the accumulation of sticky mucus induces postnasal drip, globus sensation, throat clearing, dysphagia and cough, which are prevalent symptoms in P-LPR patients [4,21,27]. In addition to the deposit of gastroduodenal content into the mucosa, the occurrence of neural reflex arc between esophagus and respiratory receptors was suspected but not yet demonstrated. This hypothesis suggests that the gastric acid stimulation of receptors within the esophagus may cause symptoms in the pharynx via neural reflex arc as well as cardiovascular and respiratory symptoms, including bradycardia and apnea [4]. 

### 4.2. Influence of Age on Physiology 

The main mechanisms of defense against laryngopharyngeal reflux involve esophageal motility/peristalsis, mucosa bicarbonate secretion and lower (LES) and upper esophageal sphincter (UES) tonicity [5,26]. The backflow of gastroduodenal content into the upper aerodigestive tract is related to esophageal dysmotility and the transient relaxation of LES and UES, which may be frequent in infants younger than 6 months and preterm infants. Indeed, younger infants report a high prevalence of gastroesophageal reflux and GERD because of the impaired esophageal peristalsis, physiological immaturity of the esophageal sphincter, and slower gastric emptying time [28,29]. The immaturity of the upper digestive tract is more important in infants born after less than 34 weeks of gestation with a GERD incidence reaching 22% of cases [30]. The intake of increased volume of liquid/milk at each feeding in this period of life and the related postprandial gastric distension and LES relaxation are additional factors supporting the high incidence of GERD and, theoretically, P-LPR in the first 6 months of life [7]. In practice, regurgitation is one of the most frequent symptoms of GERD and P-LPR in infants [12], and each episode of regurgitation is associated with the deposit of gastroduodenal enzymes into the upper aerodigestive tract mucosa, which is less protected against enzymes than esophageal mucosa [23]. 

From an evidence-based point of view, there is a limited number of studies in which authors used MII-pH monitoring in the pediatric population, while there is no study of HEMII-pH. All of the above-mentioned pathophysiological mechanisms were experienced in animal models or adults. The use of HEMII-pH may undoubtedly improve the understanding of physiological mechanisms and the differences between GERD and LPR. Precisely, infants and children with laryngopharyngeal reflux may have pharyngeal reflux events but no GERD findings at the gastrointestinal endoscopy or GERD symptoms [7]. HEMII-pH may specify the features of pharyngeal reflux events in terms of composition (gaseous, liquid, both), pH (acid, weakly acid, alkaline) and the time of occurrence (upright/daytime, supine/nighttime). 

### 4.3. Associated Conditions 

The inflammatory reaction related to the deposit of gastroduodenal enzymes into the laryngopharyngeal, nasopharyngeal or nasal mucosa may favor the development of some ear, nose and throat diseases. Thus, P-LPR was objectively identified in the following conditions: apnea and cardiorespiratory events [15,31,32,33,34,35,36], laryngomalacia [21,37,38,39], subglottic stenosis [21,40], and chronic otitis media with effusion [41,42,43,44,45,46,47,48,49,50,51,52,53,54,55] (Table 1). Precisely, infants with apnea reported a high prevalence of acid and nonacid proximal esophageal reflux events at the MII-pH with or without temporal association between reflux and cardiorespiratory events (Table 1). Regarding laryngomalacia, reflux was identified in infants and children through oropharyngeal pH study, dual-probe pH monitoring or pepsin analyses [21,37,38,39]. Note that there were no controlled studies comparing the prevalence of P-LPR in apnea, laryngolomalacia and subglottic stenosis patients vs. healthy controls (Table 1). The most exhaustive and evidence-based literature concerned the association between reflux and chronic otitis media with suppuration (Table 1). Pepsin or bile salts were found in 14% to 100% of ear secretions (Table 1). According to controlled studies, infants or children with chronic otitis media with suppurations reported higher pepsin concentrations in middle ear secretions and acid pharyngeal reflux events at the dual-probe pH study than controls [50,54]. Other studies supported a potential association between reflux and chronic rhinosinusitis [56,57,58], choanal atresia [59], asthma and chronic aspirations [60,61,62,63], but the lack of controlled study with (HE)MII-pH limits the draw of reliable conclusions.

## 5. Clinical Presentation

Infants and children with P-LPR may present a myriad of non-specific symptoms and findings. The potential associations between P-LPR and the above-mentioned ear, nose and throat conditions make the clinical presentation even more non-specific. Surprisingly, few studies investigated the prevalence of symptoms and signs associated with P-LPR with validated clinical instruments. Clinical studies with the largest number of P-LPR cases are summarized in Table 2 [61,64,65,66,67,68]. The most prevalent symptoms associated with P-LPR include breathing disorders, chronic cough, hoarseness, and postnasal drip. GERD-related symptoms include less prevalent overgrowth, especially in children [61,64,65,66,67,68]. The GERD symptoms are more prevalent in infants. Infants present more frequently a clinical picture characterized by GERD and LPR symptoms, while children report a clinical presentation closest from adults, with LPR symptoms and few GERD symptoms. 

Oral, pharyngeal and laryngeal finding prevalence were reported in two studies, in which P-LPR was confirmed with objective tools [65,69]. From a fiberoptic examination standpoint, larynx appears to be the most affected organ (Table 3), which may be related to the pseudostratified epithelium that is less resistant to pepsin aggression than the multilayer epithelium or pharynx or oral cavity. However, there would be an overestimation of laryngeal findings and an underestimation of pharyngeal LPR-signs in studies, because most investigators assessed the P-LPR signs with Reflux Finding Score (RFS), which only considers laryngeal signs [70,71]. To date, there is no large-cohort study evaluating the prevalence of oral, laryngeal and pharyngeal signs in P-LPR patients. 

In sum, a few studies investigated the clinical picture of P-LPR in infants and children with patient-reported outcome questionnaires considering otolaryngological, digestive and respiratory symptoms. The consideration of respiratory and digestive symptoms is particularly relevant in infants who have both GERD and LPR. Similar observations may be found for clinical instruments that do not include oral, pharyngeal and laryngeal signs. The development of such tools in the pediatric population is a future important step to establish the prevalence of symptoms and signs in infants and children with a documented LPR at the HEMII-pH [72,73]. The clinical tools should be adapted to patient age, considering the parent observations for infants and the higher prevalence of GERD-related symptoms in infants than children. 

## 6. Diagnostic

The past consideration of P-LPR as an extra-esophageal manifestation of GERD led some authors to use gastrointestinal (GI) endoscopy, barium contrast radiography, scintigraphy or single-probe pH monitoring for the P-LPR diagnostic. However, none of these methods may detect weakly acid or alkaline pharyngeal reflux episodes or the deposit of gastroduodenal content into the upper aerodigestive tract mucosa.

### 6.1. Multichannel Intraluminal Impedance pH Monitoring

Multichannel intraluminal impedance pH monitoring was introduced to capture weakly acidic or alkaline reflux episodes in the proximal esophagus. To date, the MII-pH profile of P-LPR infants and children was poorly investigated. Dy et al. reported that infants and children with suspected P-LPR had a similar number of proximal esophageal acid (pH < 4.0) and weakly/non-acid reflux events [64]. Mantegazza et al. observed that infants reported more weakly acid proximal esophageal reflux events than children [74]. The time (post-meals), the nature (gaseous, liquid, or mixed) and the position (upright, supine) features of occurrence of reflux events at the MII-pH have not been investigated in P-LPR. This information is, however, important for improving the therapeutic management of infants and children with P-LPR. In adults, LPR is mainly weakly acid (pH = 4.0 to 7.0) or alkaline (pH > 7.0), with an increase in the pH of the event from the low to the upper esophagus [25]. Most events are gaseous and occur daytime and upright, which may explain the lack of heartburn or regurgitations in most patients [25,75,76]. Consequently, the deposit of pepsin and other gastroduodenal enzymes into the upper aerodigestive tract mucosa occurs after the meals through the transient relaxations of LES and UES [25,77]. The knowledge of the LPR profile at the HEMII-pH may contribute to the development of more personalized therapeutic approaches [78,79], which may include diet and lifestyle modifications, PPIs (acid LPR or GERD), alginate or magaldrate (weakly acid/alkaline reflux events).

In sum, the profile and the features of P-LPR at the HEMII-pH are still unknown in both infants and children because the authors used MII-pH, and not HEMII-pH. MII-pH is currently the most used approach for the objective diagnostic of P-LPR, but physicians have to take into consideration that only some proximal reflux events reach the pharynx. Indeed, Ulualp et al. reported in a cohort of children with acid P-LPR that 6/9 proximal esophageal reflux episodes reached pharynx [80]. From an evidence-based approach, the consideration of proximal esophageal reflux events for the P-LPR diagnostic may be insufficient due to the lack of objectification of pharyngeal event and the potential lack of related deposit of gastroduodenal enzymes.

### 6.2. Pepsin Saliva Measurement

The development of less invasive objective methods for the LPR diagnosis makes particularly sense in infants and children who do not frequently tolerate the 24 h pH-testing probe [21,81]. Nowadays, the usefulness and the accuracy of pepsin saliva detection (PepTest^®^, RDBiomed, Hull, UK) have been poorly investigated in infants and children with P-LPR. Haddad et al. observed that pepsin was detected in 48% of infants and children with a positive GERD diagnosis at the MII-pH. The pepsin A was 43% sensitive and 50% specific in predicting an abnormal impedance result. Among pepsin A positive samples, 72% of samples corresponded to a gastroesophageal reflux episode. The authors reported that pepsin peak levels were significantly correlated with acid GERD [82]. The usefulness of pepsin measurement in pediatric patients was supported by Klimara et al. in a controlled study where authors compared pepsin saliva measurements between pediatric patients with laryngomalacia and healthy controls [38]. These authors detected pepsin in the saliva of 81% of infants with laryngomalacia and suspected P-LPR, while controls reported a positive pepsin rate in 12% [38]. The accuracy of pepsin saliva detection was investigated in infants and children with GERD undergoing 24 h MII-pH [83]. The authors collected several saliva samples (before catheter placement, before and 30 min after each of three meals, and upon awakening), and they reported that 85.6% of GERD patients have at least one positive saliva pepsin test compared with 9.3% of controls [83]. Interestingly, they observed a significant positive correlation between the frequency of pepsin-positive samples and the reflux symptom index [83].

To date, the pepsin saliva measurement is not ready for clinical application for many reasons [84]. First, the pepsin saliva concentration may significantly vary throughout the testing day [85,86,87]. This variation may be attributed to both physiologic and pathologic modifications of esophageal motility and sphincter tonicity, which may be related to lifestyle, meal composition and autonomic nerve dysfunction (anxiety/stress) [85,86,87,88]. Second, the best time of saliva collection, which may be defined as the saliva collection associated with the highest accuracy, sensitivity and positive predictive value pepsin test, remains unknown. The fasting collection of saliva appears to be adequate, but studies were only conducted on LPR adults [85,86,87,88,89]. Third, as recently suggested, the mucosa injury may be related to other gastroduodenal enzymes, such as bile acids, trypsin or elastase [90,91]. Thus, the only consideration of saliva pepsin detection as a diagnostic approach may bias the detection of P-LPR.

In sum, the detection of gastroduodenal enzymes into the infant or child saliva is an interesting noninvasive approach that should provide the profile of P-LPR in terms of involved enzymes in the inflammatory process underlying LPR symptoms and findings. The determination of the enzyme profile and the knowledge of their pH activity may theoretically indicate the use of PPIs or alginate in some pediatric patients. In practice, infants and children with a prominence of saliva enzymes that are active in weakly acid or alkaline pH (e.g., bile salts, elastase) should benefit from alginate and not PPIs, which increase the pH of reflux events. Thus, some enzymes, e.g., elastase, trypsin or bile salts, are activated in alkaline environments and may therefore lead to mucosa injuries and inflammation from the use of PPIs [91]. Alginate and magaldrate form a raft floating over gastric contents that can be maintained within the stomach for up to 4 h. Alginate is endowed with bio-adhesive potential, which is a property due primarily to its polymer chain length and ionizable groups that provide a protective biofilm on the mucosa of esophagus and, potentially, upper aerodigestive tract [92].

## 7. Therapeutic Strategies

Therapeutic strategies for P-LPR include diet and lifestyle changes, medical treatment or surgery. From a cost-effective standpoint, the first therapeutic step has to be based on lifestyle and diet changes. Lifestyle and diet modifications may include the reduction in foods and beverages associated with sphincter tonicity and esophageal motility impairments, the suppression of reflux triggers, and the management of autonomic nerve dysfunction, which is commonly associated with anxiety or stress management [93,94]. Among diet modifications, the utility of GERD recommendations, such as thickening feeds or avoiding cow’s milk protein, remains undemonstrated in P-LPR regarding the lack of studies including infants or children with a positive diagnostic at the HEMII-pH. Similar observations may be made regarding the influence of type of milk on the occurrence of pharyngeal reflux events, because previous studies included GERD patients [95,96,97]. Smaller and frequent meals as well as sleep positioning (elevating the head of the bed) may provide benefit to infants with GERD and ‘reflux extension’ into the upper aerodigestive tract [6,98], but the utility of these approaches is still not demonstrated in children with only P-LPR [4].

If lifestyle and diet modifications are insufficient in resolving P-LPR symptoms, a medical approach might be considered. The medical treatment of P-LPR was long-standing based on the use of histamine (H2) blockers or PPIs. PPIs and histamine (H2) blockers bind irreversibly to active proton pumps and increase the pH of gastroesophageal and esophago-pharyngeal reflux events without influencing the number and duration of events [4,99]. In adults, the superiority of PPIs *over* placebo is not demonstrated [100]. The success rate of PPI therapy is significantly lower that of PPI therapy in GERD patients [101]. To date, there is no randomized controlled trial comparing the effectiveness of PPIs over placebo in pediatric population. In recent prospective studies, Li et al. reported a success rate of PPIs in 53% of P-LPR patients [66], while Jadcherla et al. reported 33% of symptom improvement in young infants [102]. The poor efficacy of PPIs in the P-LPR treatment and the lack of confidence by practitioners were supported in the recent survey of the American Society of Pediatric Otolaryngology. Thus, the authors reported that 37% of otolaryngologists would not prescribe oral PPIs in neonates, with 50% not prescribing IV PPIs. PPIs were prescribed by only 10% and 60% of otolaryngologists as first or second/third-line treatment for infants aged from 10 weeks to 1 year, respectively [103].

Despite the lack of study using HEMII-pH, weakly acid or alkaline reflux events in the proximal esophagus will be weakly acid or alkaline in the pharynx. Thus, from an evidence-based standpoint, the high prevalence of weakly acid or alkaline reflux events at the MII-pH may make the consideration of PPIs or histamine (H2) blockers as first-line single medication in infants or children with P-LPR outdated. In infants with both GERD and P-LPR, acid-suppressive therapy should be used most likely in the context of symptoms that suggest erosive esophagitis [96]. Future controlled studies comparing diet/lifestyle modifications vs. PPIs vs. alginate vs. PPIs and alginate as empirical therapeutic approaches are needed to determine the place of medication in the management of infants and children. From a personalized medicine point of view, future treatments of infants or children should consider the type of P-LPR and their characteristics at the HEMII-pH (types and composition of LPR, time of occurrence, etc.). Regarding fundoplicature, there is no evidence about a potential benefit on P-LPR.

## 8. Conclusions

Many gray areas persist in P-LPR. The lack of international guidelines for the diagnostic of P-LPR, the unavailability of HEMII-pH and the poor acid-suppressive therapeutic response are all issues requiring future investigations. Future controlled studies using HEMII-pH and enzyme measurements in ear, nose or throat fluids may clarify the epidemiology of P-LPR according to age and its association with many otolaryngological conditions.

## Figures and Tables

**Table 1:** Laryngopharyngeal reflux detection in ear, nose and throat conditions.

Condition	Population		Reflux identification	References
Apnea	Infants	Apnea was associated with high rate of	MII-pH monitoring	Pavic, 2021 [15]
		acid and nonacid reflux events.		
	Infants	Reflux is associated with cardiorespiratory	MII-pH monitoring	Nobile, 2019 [31]
		events in 11% of cases.		
	Infants	There is a temporal association between nonacid reflux events and apnea events.	MII-pH monitoring	Cresi, 2017 [33]
	Preterm infants	The frequency of apnea is increased after nonacid reflux events.	MII-pH monitoring	Corvaglia, 2011 [28]
	Preterm infants	Reflux is associated with a high risk of apnea	MII-pH monitoring	Nunez, 2011 [32]
	Preterm infants	GERD is not associated with cardiorespiratory event prolongation or severity.	MII-pH monitoring	Di Fiore, 2010 [35]
	Infants	Reflux events are prevalent in infants with apnea without reporting a temporal relationship.	MII-pH monitoring	Peter, 2002 [36]
Laryngomalacia	Children	Pharyngeal reflux events were prevalent in	Oropharyngeal pH testing	Messalam, 2016 [21]
		children with laryngomalacia.		
	Infants	Pepsin saliva was more frequently detected in laryngomalacia infants than controls.	Pepsin saliva measurement	Klimara, 2020 [38]
	Infants	Pepsin was detected in lavage laryngomalacia of 80% of infants and was absent in controls.	Pepsin lavage laryngo-	Luebke, 2017 [39]
			malacie measurement	
	Infants	Reflux was the primary cause of airway compromise or a cofactor exacerbating preexisting neurologic/anatomic abnormality.	Dual-probe pH testing	Mattheuws, 1999 [37]
Subglottic stenosis	Children	Pharyngeal reflux events were prevalent in	Oropharyngeal pH testing	Messalam, 2016 [21]
		children with subglottic stenosis.		
	Infants and children	Reflux events were prevalent in patients with subglottic stenosis.	MII-pH monitoring	Hart, 2014 [40]
Otitis media	Infants and children	Pepsin was detected in otitis secretions of 77% of infants or children.	Pepsin otitis media	Samuels, 2022 [41]
			secretion measurements	
	Infants and children	67.9% of children with middle ear effusion had weakly acid proximal esophageal reflux events.	MII-pH monitoring	Gorecka, 2016 [42]
	Infants and children	Patients with chronic otitis media reported higher prevalence of acid pharyngeal	Dual-probe pH testing	Martines, 2015 [43]
		events in the pH study.		
	Children	Pepsin was found in 32% of middle ear otitis secretions.	Pepsin in ear secretions	Formanek, 2015 [44]
	Children	Pepsin was found in 70% of children with	Pepsin in ear secretions	Luo, 2014 [45]
		chronic otitis media effusion.		
	Infants and children	Pepsin was found in 50% of children with	Pepsin in ear secretions	O’Reilly, 2014 [46]
		chronic otitis media effusion.		
	Children	All children with chronic otitis media with	Dual-probe pH-testing	Abdel-Aziz, 2013 [47]
		effusion had positive acid LPR at the pH study.		
	Children	Nasopharyngeal acid reflux was detected in 20% of patients with chronic otitis	Dual-probe pH-testing	Aydin, 2011 [48]
		media.		
	Infants and children	Bile salts were found in 42% of middle ear	Bile salts	Klokkenburg, 2009 [49]
		secretions of patients with chronic otitis media.		
	Infants and children	Patients with chronic otitis media and controls had 14% and 7% positive pepsin in ear secretions.	Pepsin in ear secretions	He, 2007 [50] *
	Infants	Pepsin was found in 58% of children with chronic otitis media with effusion.	Pepsin in ear secretions	Crapko, 2007 [51]
	Infants and children	Pepsin was found in 100% of ear secretion	Dual-probe pH-testing	Abd El-Fattah, 2007 [52]
		samples.	Pepsin in ear secretions	
	Infants	Pepsin was detected in 73% to 77% of ear	Pepsin in ear secretions	Lieu, 2005 [53]
		secretions of patients with chronic otitis media.		
	Children	Acid pharyngeal reflux events were found in 48% vs. 8% of patients with chronic otitis media and controls, respectively.	Dual-probe pH testing	Keles, 2004 [54]*
	Children	27% of patients with chronic otitis media with effusion had proximal esophageal reflux events.	Dual-probe pH testing	Rozmanic, 2002 [55]

* controlled studies. Abbreviations: GERD = gastroesophageal reflux disease; MII-pH = multichannel intraluminal impedance pH monitoring; LPR = laryngopharyngeal reflux.

**Table 2:** Symptoms of Pediatric Laryngopharyngeal Reflux.

References	Patients (N)	Diagnosis	Symptoms	(%)
Kosec, 2020 [61]	89 infants/children	MII-pH	Epigastric pain	17%
	F/M: 56/33		Nausea	16%
	Age (md): 12 yo		Regurgitations	11%
			Tasting acid in mouth	3%
Dy, 2016 [64]	50 infants/children	MII-pH	Chronic cough	84%
	F/M: 16/34		Asthma symptoms	71%
	Age (me): 9 yo		Croup	48%
			Ear infections/symptoms	24%
			Sinus infections/symptoms	16%
Baudoin, 2014 [65]	50 infants/children	Dual-probe	Asthma symptoms	33%
	F/M: N.P.	pH testing	Chronic cough	28%
	Age (md): 11 yo		Dysphonia	7%
			Chest pain	6%
Li, 2014 [66]	62 infants/children	Dual-probe	Hoarseness	90%
	F/M: N.P.	pH testing	Postnasal drip	77%
	Age: N.P.		Dysphagia	74%
			Abdominal/chest pain	73%
			Throat clearing	64%
			Chronic cough	56%
			Globus	48%
Andrews, 2013 [67]	63 infants/children	Oropharyngeal	Nasal obstruction	76%
	F/M: N.P.	pH study	Chronic cough	54%
	Age: 6 mo-17 yo		Postnasal drip	40%
			Otalgia	30%
			Halitosis	18%
			Sore throat	16%
			Loss of appetite	14%
			Rhinosinusitis symptoms	10%
			Stomachache	10%
Greifer, 2012 [68]	63 infants/children	MII-pH	Asthma/cough	59%
	F/M: 24/39		Hoarseness	22%
	Age (me): 7 yo		Vocal cord nodules	14%
			Pharyngitis	5%

Abbreviation: F/M = female/male; md = median; me = mean; MII-pH = multichannel intraluminal impedance pH monitoring; mo = month; NP = not provided; yo = years old.

**Table 3:** Signs of Pediatric Laryngopharyngeal Reflux.

References	Patients (N)	Reflux Diagnostic	Signs	(%)
Baudoin, 2014 [65]	50 infants/children	Dual-probe	Vocal fold edema	63%
	F/M: N.P.	pH testing	Oropharyngeal wall or	54%
	Age (md): 11 yo		posterior larynx granulation	
			Vocal fold nodules	26%
			Oropharyngeal wall granulations	14%
			Posterior laryngitis	10%
			Vocal nodules	1%
Galli, 2020 [69]	35 infants/children	MII-pH	Arytenoid erythema	100%
	F/M: 11/24		Vocal mucosa edema	76%
	Age: 2 mo-16 yo		Pharyngeal wall granulations	51%
			Laryngeal edema	50%
			Posterior commissure edema	34%
			Tracheal hyperemia	30%
			Subglottic edema	5%

Abbreviation: F/M = female/male; MII-pH = multichannel intraluminal impedance-pH monitoring; mo = month; NP = not provided; yo = years old.

## Data Availability

Not available.
